# Understanding Biofilm Formation in Ecotoxicological Assays With Natural and Anthropogenic Particulates

**DOI:** 10.3389/fmicb.2021.632947

**Published:** 2021-07-01

**Authors:** Elena Gorokhova, Asa Motiei, Rehab El-Shehawy

**Affiliations:** Department of Environmental Science (ACES), Stockholm University, Stockholm, Sweden

**Keywords:** actinobacteria, bacterial assemblages on microplastic, BALOs, *Daphnia magna*, ecotoxicological testing, microbiome, particle aggregation, plastic debris

## Abstract

Fossil-made polymers harbor unique bacterial assemblages, and concerns have been raised that ingested microplastic may affect the consumer gut microbiota and spread pathogens in animal populations. We hypothesized that in an ecotoxicity assay with a mixture of polystyrene (PS) and clay: (1) microbiome of the test animals inoculates the system with bacteria; (2) relative contribution of PS and the total amount of suspended solids (SS) select for specific bacterial communities; and (3) particle aggregation is affected by biofilm community composition, with concomitant effects on the animal survival. Mixtures of PS and clay at different concentrations of SS (10, 100, and 1000 mg/L) with a varying microplastics contribution (%PS; 0–80%) were incubated with *Daphnia magna*, whose microbiome served as an inoculum for the biofilms during the exposure. After 4-days of exposure, we examined the biofilm communities by 16S rRNA gene sequencing, particle size distribution, and animal survival. The biofilm communities were significantly different from the *Daphnia* microbiota used to inoculate the system, with an overrepresentation of predatory, rare, and potentially pathogenic taxa in the biofilms. The biofilm diversity was stimulated by %PS and decreased by predatory bacteria. Particle aggregate size and the biofilm composition were the primary drivers of animal survival, with small particles and predatory bacteria associated with a higher death rate. Thus, in effect studies with solid waste materials, ecological interactions in the biofilm can affect particle aggregation and support potentially harmful microorganisms with concomitant effects on the test animals.

## Introduction

Fueled by recent interest in microplastic pollution research, the interactions of living matter with solid materials, with particular focus on anthropogenic materials, has become a subject of great interest. However, when testing the effects of particulates in biota, the pivotal role of surface chemistry, and other components, both living (e.g., microorganisms) and non-living (e.g., dissolved organics), has been understudied. Despite frequent suggestions that particle behavior and physicochemical properties govern the interactions between biota and test particles (Anbumani and Kakkar, 2018; Rist and Hartmann, 2018), experimental data on these parameters, interactions and their contribution to the observed responses are limited. In reviews and synthesis papers, the ecological impact of microplastic pollution is frequently suggested to be a function of the biofilms colonizing plastics in the environment – termed the “Plastisphere” (Rogers et al., 2020). However, to date, most biological tests on xenobiotic solids, including animal experiments, have been performed with poorly defined materials, uncontrolled exposure levels, and insufficient control of the microorganism populations in the test systems, so that some of the data generated have limited value for understanding the effects and comparing particle toxicities across studies (Bucci et al., 2020).

Once in aquatic ecosystems, plastics, like any other materials, are quickly colonized by diverse microbes forming biofilms (Rogers et al., 2020). These biofilm communities on plastics often differ from those in the surrounding water and other substrates (Ogonowski et al., 2018). Moreover, plastic surfaces inhabited by biofilms undergo various physicochemical transformations (McGivney et al., 2020), resulting in changing particle capacity to sorb chemicals and aggregate with other particulates. In turn, aggregation affects particle size distribution (PSD) in the environment, altering the plastics uptake by consumers with size-selectivity toward food particles. On the other hand, the biofilms on the aggregates can improve animal nutrition, particularly at low food availability (Kirk, 1992), and establish/strengthen pathways for channeling bacterial production to non-bacterivores in the food webs (Rogers et al., 2020).

The exposure to grazers and their gut microbiota, followed by the substrate-driven selection in the biofilm communities, make microplastics a likely substrate for fostering unique assemblages of taxa, including pathogens and bacteria resistant to antimicrobials and various environmental contaminants. The main concerns are that microplastics can act as both a substrate for enhanced selection and a vector for spreading these taxa in the food webs, both horizontally (i.e., within and between filter-feeder populations) and vertically (e.g., from prey to a predator), which is frequently suggested to be a potential threat to environmental and human health.

Biofilm formation and biofilm-mediated effects on the consumers are also relevant for experimental systems in effect studies. Moreover, controlled experiments can be used to evaluate the plausibility of the microbiome-mediated biofilm effects of solid waste materials, including microplastics. When animals are exposed to test particles in a closed system of an ecotoxicological assay, biofilm-forming bacteria can be introduced with the test particles, labware, media, and animal food. Moreover, unless the germ-free animals are used in the assay, some microorganisms are always introduced with the test organism microbiota. During the exposure, these microorganisms can establish biofilm communities on the test particles, and these communities will likely differ from the inoculum because of the substrate selectivity and generally low nutrient levels, with a possible proliferation of rare taxa that are not beneficial for the host. Further, the biofilm quantities and composition can influence the bioavailability of the test material via aggregation (Motiei et al., 2021) while also increasing the abundance of the non-beneficial microbiota. All these factors may affect the response measured in the test organism.

Here, we hypothesized that in ecotoxicity assay with a mixture of suspended solids (SS) composed of microplastics and other particulates, the test organism microbiome inoculates the system with biofilm-forming bacteria (H1). Moreover, microplastics relative contribution and the total amount of suspended solids would affect the biofilm community composition (H2). Finally, biofilm community composition would affect particle aggregation, with concomitant effects on the animal survivorship (H3). We tested these hypotheses experimentally using *Daphnia magna*, a model test species with a well-studied microbiome, exposed to a mixture of kaolin clay and microplastics.

## Materials and Methods

We capitalized on the material collected as a part of the experimental study reported elsewhere (Motiei et al., 2021). In the latter study, *D. magna* was exposed to a mixture of UV-aged fragmented polystyrene and kaolin clay, with and without biofilms that were generated using natural bacterioplankton communities, to evaluate survivorship drivers. In this experiment, we found that suspended solids that were not exposed to the natural bacterioplankton had developed measurable bacterial biofilms, with the daphnids being the only possible source of the microorganisms. Here, to further understand the capacity of the microbiome to establish biofilms in the ecotoxicological test system, the role of the bacterial biofilm in the particle aggregation during the exposure, and the possibility of transferring microbiome components within and between animal populations by the particulates, we conducted bacteria community analysis using the daphnids collected at the start of the exposure and the particle suspensions collected at the end of the exposure.

### *Daphnia magna* Culture

As a test species, we used the freshwater cladoceran *D. magna*, a model organism in aquatic ecology and ecotoxicology. It has been extensively used in the effect studies with both inorganic suspended solids (Arruda et al., 1983; McCabe and O’Brien, 1983; Antunes et al., 2007; Ogonowski et al., 2016) and microplastics (Ogonowski et al., 2016; Aljaibachi and Callaghan, 2018; Gerdes et al., 2019), but also to study the ability of filter-feeders to change PSD and composition of particulates in suspension (Filella et al., 2008). All experimental animals originated from the same clone (Clone 5; The Federal Environment Agency, Berlin, Germany) cultured in M7 media, often referred to as reconstituted lake water (OECD, 2012) and fed *ad libitum* with axenically grown green algae (*Raphidocelis subcapitata* and *Scenedesmus spicatus*) three times a week.

### Test Materials and Test Mixtures

The test particle stock suspensions were prepared using kaolin clay (Sigma-Aldrich, K7375; size range 2–40 μm) and UV-aged polystyrene microplastics (PS; size range: 3–20 μm) in the M7 medium; see Supplementary Text 1 and Supplementary Table 1 for details on the preparation of the particle suspension stocks and their characteristics. Polystyrene often contributes to plastic debris in the environment, where abiotic and biotic factors contribute to its breakdown resulting in weathering and fragmentation (Barnes et al., 2009); this is why we used UV-aged material for this experiment.

The test mixtures based on nominal concentrations of kaolin and PS were prepared in batches with 0, 10, 20, 40, and 80% microplastics contribution to the total amount of suspended solids of 10, 100, and 1000 mg/L; each replicate was treated separately. Each test suspension was then transferred to a 50-mL polypropylene centrifuge tube and used as an incubation vial.

### Experimental Setup and Procedures

The factorial experimental design was applied with three test concentration of SS (10, 100, and 1000 mg/L) and varying percentage of polystyrene in the particle mixture (%PS; 0, 10, 20, 40, and 80%) as the experimental factors. In total, 15 treatment combinations (%PS × SS concentration) and two kinds of control were used (Table 1); each treatment combination was conducted in seven replicates. As a procedural control for survivorship, we used laboratory-reared daphnids incubated in the M7 medium (10 individuals per replicate, 7 replicates) at the same experimental conditions as the test treatments, with no particles added. As a negative control for bacteria contamination, animal-free incubations were set using a mixture with 80% microplastics at 1000 mg/L three replicates; these incubations were used to confirm that no measurable quantities of the biofilm on the suspended particulates were produced in the absence of daphnids during the exposure time (Motiei et al., 2021).

**TABLE 1:** The experimental setup.

**Treatment**	**Experimental media**	**Daphnids**	**SS concentration, mg L^–1^**
Procedural control	M7	Yes	0
Negative control [80 %PS]	M7 + Kaolin + PS	No	1000
0 %PS	M7 + Kaolin	Yes	10, 100, 1000
20 %PS	M7 + Kaolin + PS	Yes	10, 100, 1000
40 %PS	M7 + Kaolin + PS	Yes	10, 100, 1000
80 %PS	M7 + Kaolin + PS	Yes	10, 100, 1000

*Daphnia magna (neonates, 10 individuals per replicate) were exposed to test particles, a mixture of weathered polystyrene (PS; contribution 0–80% by mass) and kaolin at total suspended solid concentrations of 10, 100, and 1000 mg L^–1^. Procedural control (daphnids only, no added particles) and negative control (no daphnids) were included. Each treatment included seven replicates, except for the negative controls, where three replicates were used. Standard growth media for *Daphnia* culture (M7) was used as the exposure media.*

The exposure experiment was designed according to Gerdes et al. (2019). In brief, 10 neonates (<24-hour old) of *D. magna* were introduced in each tube containing either a test mixture (%PS × SS) or a control. All tubes were mounted on a plankton wheel rotating at 0.5 rpm in a thermo-constant room at 21°C with a light: dark cycle of 16:8 h; at this rotation speed, no particle sedimentation was observed. The test was terminated after 96 h.

Upon the experiment termination, the daphnids were removed from the tubes, and mortality was recorded in each replicate (OECD, 2004). Each %PS × SS treatment combination was assigned to either PSD analysis (three replicates) or biofilm analysis (three replicates); one replicate was kept as a reserve. The negative controls were used for the biofilm analysis only. The samples designated for biofilm analysis (i.e., 16S rRNA NGS analysis and quantity of the biofilm) were collected by filtering the test suspension (50 mL) on MILLIPORE HA filters (47 mm, 0.45 μm); the filters were stored at −80°C in Eppendorf tubes until the analysis. To measure quantities of the particle-associated biofilm, we used DNA concentration as a proxy for the microorganism abundance. The DNA samples were further used to characterize bacterial communities by pooling three replicates within each %PS × SS treatment combination (15 samples in total) for 16S rRNA gene sequencing. Also, to determine the composition of the bacterial communities introduced in the system, three *Daphnia* neonate samples (five ind./sample) were processed in the same manner as the filter samples. Finally, to control for bacteria contamination, 200 μl of particle stock suspensions were subjected to the DNA extraction protocol; however, no measurable DNA quantities were obtained.

#### Particle Aggregation Analysis

To analyze PSD (1–100 μm) in the stocks and test mixtures, we used Spectrex Laser Particle Counter (Spectrex, PC-2000, Redwood City, CA, United States); see Supplementary Text 1 for details. Depending on the SS levels, the experimental suspensions were diluted up to 1000-fold to comply with the instrument linear dynamic range; particle-free deionized water (<10 counts/mL) was used for dilution. The size spectra were analyzed with Gradistat software 8.0 (Blott and Pye, 2001) following the computational method of Folk and Ward (1957). As most of the size spectra deviated significantly from the normal distribution, we used a median particle size (D_50_, μm) to represent the most common aggregate size of the particle mixture.

#### Genomic DNA Isolation

Total DNA was extracted from the filters using 10% Chelex (Straughan and Lehman, 2000); negative animal-free controls and blanks for sequencing were processed simultaneously. The DNA concentrations were measured using Quant-iT PicoGreen dsDNA Assay kit (ThermoFisher, Waltham, MA, United States), and quantified fluorometrically with a Tecan Ultra Spectro Fluorometer (PerkinElmer, Waltham, MA, United States), with excitation and emission set at 480 and 530 nm, respectively. The concentration measurements were used to normalize the DNA amount in PCR reactions and to represent the bacteria quantities in the system. As a proxy for biofilm relative contribution to suspended solids, or biofilm thickness, we used mass-specific DNA concentration (DNA/SS), representing the amount of DNA normalized to the nominal mass of suspended solids in the system. Here, the assumption was that DNA associated with the particulates is entirely of bacterial origin and represents living bacteria on the particle surface.

#### Library Generation and Bacterial 16S rRNA Sequencing

In preparation for the 16S rRNA gene sequencing, the DNA samples were purified with AMPure XP beads (Beckman Coulter, Brea, CA, United States) following the manufacturer’s instructions. Due to the low DNA concentrations in the biofilm samples, a two-steps PCR amplification was performed following the protocol of Motiei et al. (2020) and using primers Bakt_341F (CCTACGGGNGGCWGCAG) and Bakt_805R (GGACTACHVGGGTWTCTAAT), which amplify V3–V4 region of the 16S rRNA gene of bacteria (Herlemann et al., 2011). No PCR amplicons were obtained from the negative controls and these samples were not submitted for sequencing. The PCR amplicons were subjected to purification and library preparation using 5 μl of equimolar amounts of the purified PCR products. Quality control was performed on an Agilent 2100 BioAnalyzer using high sensitivity DNA chip. Libraries were denatured using 0.2 N NaOH and sequenced using the MiSeq Illumina system (2 × 300 bp paired-end) with the v3 reagent kit (600-cycles), following the manufacturer’s instructions.

#### 16S rRNA Sequence Analysis

Illumina software v. 2.6.2.1 was used for demultiplexing and removal of indexes and primers according to the standard Illumina protocol. Following demultiplexing, removal of tags and primers, the reads were processed using the DADA2 package (version 1.6) as implemented in the R statistical software v. 3.4.2 (R Core Team, 2016) that detects and removes low-quality sequences and merges paired-end reads to generate amplicon sequence variants (ASVs). The sequences were trimmed 60 bp downstream of the forward primer and 100 bp downstream of the reverse primer. Sequence quality control was performed by identifying and removing chimeric sequences and those containing ambiguous bases. The resulting sequences were classified with the SILVA taxonomy (Silva Ribosomal RNA database; version v.132), and eukaryotic ASVs were removed. All raw sequence files, including sequencing controls, are available from the NCBI Short Read Archive (SRA) database (BioProject ID: PRJNA674527 for the biofilm samples and BioProject ID: PRJNA560134; samples A65, A67, and A69 for *D. magna*).

Illumina MiSeq sequencing of all amplicons resulted in a total of 95 466 high-quality filtered reads in the *Daphnia* and biofilm samples (Supplementary Figure 1), with a mean read depth per sample of 5 303 sequences and a total ASV number of 3 028 (428 with ≥2 counts). Data filtering was done using a minimum count of 2 and 10% prevalence to remove low quality or uninformative features. Due to the high variability in the sequence libraries (Supplementary Figure 1), the data were normalized for diversity analysis using rarefaction curves. Rarefaction curves and Zhang–Huang’s coverage estimator were calculated from ASV abundances using functions supplied by the *vegan* and *entropart* R packages. The rarefaction curves (Supplementary Figure 2) were obtained by subsampling the ASV table with step increments of 1000 sequences and 100 iterations at each step.

### Data Analysis and Statistics

#### Analysis of Microbial Diversity, Community Structure, and Composition

Alpha diversity at the family level was assessed using the Chao1, Shannon–Wiener, Fisher’s alpha and Simpson indices at the ASV level. Significant differences in the diversity between the sample types were tested with Welch’s *t*-test accounting for unequal variance and unbalances sample size. Beta diversity was determined based on the Bray–Curtis index distance method, and principal coordinate analysis (PCoA) plots were generated. Permutational multivariate analysis of variance (PERMANOVA) was used to analyze beta diversity. In addition, a one-way ordered analysis of similarity (ANOSIM) was used to give an insight into the degree of separation between the tested groups of samples. ANOSIM tests the null hypothesis that the average rank similarity between samples within a group is the same as the average rank similarity between samples belonging to different groups. To test the hypothesis of equal within-group dispersion PERMDISP analysis was also conducted using Bray–Curtis index distance method to supplement results of the PERMANOVA and ANOSIM; *microbiome* (version 1.1.2) R package was used for these analyses.

To identify which taxa were responsible for the observed differences in the diversity and community structure, we first explored the taxonomic structure of biofilm and microbiota communities using relative abundance of taxa contributing >1% to the communities. Then, the core microbiomes of the *Daphnia* and biofilm communities, defined as a set of bacteria consistently present in the samples, were constructed using the *microbiome* package; the prevalence was set at 20% and detection threshold at 0.01%. The co-occurrence network based on Sparse Correlations of species with differential abundance was constructed using SparCC designed to deal with compositional data (Friedman and Alm, 2012); R package *SpiecEasi*, version 1.0.7, was used. SparCC does not depend on any particular distribution and performs well with low-diversity and high sparsity, making it suitable for our data set. Bootstrapped tables were generated and used to calculate SparCC correlations. Pseudo *p*-values were calculated as the proportion of simulated permutated data sets with a correlation at least as extreme as that computed for the original dataset; a correlation threshold of 0.3 was used. Then, the corrected two-tailed *p*-values were calculated by the Benjamini–Hochberg method, and the edges in the network were filtered with *p* < 0.01.

#### Effects of the Experimental Factors on Daphnia Mortality and Biofilms

Generalized linear models (GLMs) with normal error structure and log-link or identity functions (Statistica 13.0, TIBCO Software, Inc., Palo Alto, CA, United States) were used to evaluate predictors of *Daphnia* mortality (Mortality, %), particle aggregation (D_50_), biofilm thickness (DNA/SS), and biofilm diversity and relative abundance of influential taxonomic groups. We also tested whether DNA amount in the system (DNA) was driven by *Daphnia* mortality because at least part of the DNA in the system could have been originated from the host.

The GLM analysis started with the identification of the primary drivers of mortality. Further, we hierarchically identified drivers for each significant mortality predictor using the same approach. In all GLMs, SS and %PS were included as potential predictors because these were the main experimental factors. Other predictors included variables related to the PSD and biofilm diversity indices and relative abundance of the three dominant families present in the biofilms (Bdellovibrionaceae, Nocardiaceae, and Mycobacteriaceae); see Supplementary Table 3 for the full list of predictors considered in the GLM analysis. These variables were relevant for the mortality dynamics because: (1) bacteria can provide the animals with some nutrition and alleviate mortality, (2) biofilm community structure can affect *Daphnia* microbiota, and (3) formation of large aggregates decreases particle number concentrations for smaller and more harmful particles (Motiei et al., 2021). The aggregate size and topology, as well as biofilm thickness, may also affect bacterial diversity. Therefore, their significance as covariables in the mortality and diversity models was tested.

Akaike information criterion (AIC) was used to optimize the number and combination of predictive variables using the Model Building Module in Statistica. The Wald statistic was used to evaluate whether the regression coefficients are statistically significant (*p* < 0.05). Model goodness of fit was checked using deviance and Pearson χ^2^ statistics, and model residual plots were assessed visually to exclude remaining unattributed structure indicative of a poor model fit.

In all models, the mortality values were Box–Cox transformed to improve homogeneity and approach the normal distribution of the model residuals. All predictors were transformed to their *z* scores and centered, thus enabling interpretation of main effects when interaction terms were significant. Meaningful interaction effects were first included in all models but omitted if found non-significant.

To visualize the relations in the biofilm with experimental factors (SS, %PS), properties of the particle mixtures (D_50_, Sorting), and biofilm quantities (DNA, DNA/SS), we used principal component analysis (PCA). PCA biplot was based on the relative contributions of the bacterial genera (>1%) to the biofilm community in the samples grouped by SS treatment; PC1 and PC2 were used to plot the results.

## Results

### Biofilm Communities in the Inoculum and the Particle Mixture

The average coverage was 90.1% ± 2.4, indicating that the sequencing effort was sufficient to describe the vast majority of bacterial communities in the samples. The total ASV number per sample was 3501–6022 for the *Daphnia* microbiome and 83 to 20025 for the biofilm samples (Supplementary Figure 1). For the daphnid samples, the rarefaction curves reached the saturation level, whereas samples recovered from the filters did not reach an asymptote at the global scale, suggesting that some or many taxa have not yet been sequenced (Supplementary Figure 2).

**FIGURE 1:**
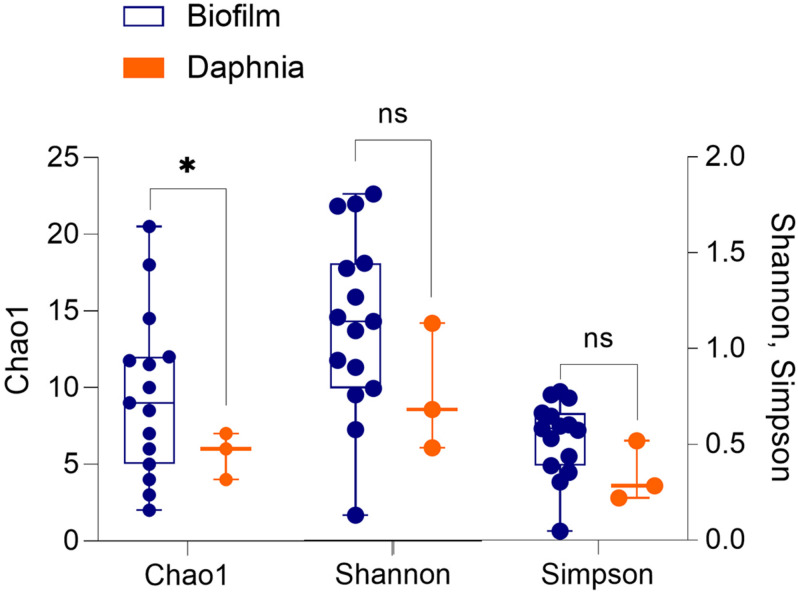
Alpha diversity indices (Chao1, Shannon–Wiener, and Simpson) in the biofilms associated with the particulate matter (*n* = 15) and in the microbiota of *Daphnia magna* used as an inoculum (*n* = 3). The groups were compared using Welch’s *t*-test (unequal variance *t*-test); *p* < 0.05: *; *p* > 0.05: ns. See Supplementary Table 2 for the statistical output.

**FIGURE 2:**
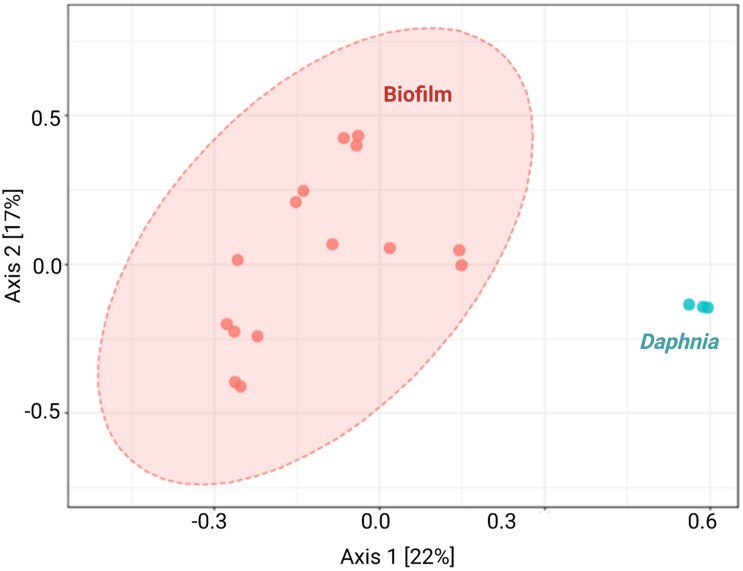
Principal coordinate analysis by sample type. Unweighted UniFrac principal coordinate analysis (PCoA) shows variation in microbiome composition between biofilms associated with the particulate matter (*n* = 15) and *Daphnia magna* used as an inoculum (*n* = 3). Each data point denotes an individual sample; analyses were conducted using *microbiome* R package.

The diversity analysis corroborated the rarefaction curve appearance by showing higher variability in alpha diversity indices in the biofilm than in the daphnid samples, and for Chao1, a significantly higher diversity in the biofilm (Figure 1 and Supplementary Table 2). Using PERMANOVA, the matrices with beta diversity measures at the family level showed significant differences in composition between the biofilm and daphnid samples (*F* = 4.0342; *R*^2^ = 0.20; *p* < 0.002; Figure 2). The unweighted UniFrac PCoA revealed a clear separation between the sample groups (Figure 2). The first three components together explain more than 50% of the variability between the samples. ANOSIM results confirmed the PERMANOVA outcome, indicating a very high separation between the groups, suggested by R statistic (*R* = 0.93; *p* < 0.002). Finally, PERMDISP confirmed that the biofilm communities were significantly more dispersed than the microbiomes (*F* = 31.04; *p* < 0.0001). Thus, the bacterial communities inhabiting test particles in the assay were significantly different from the *D. magna* microbiome used to inoculate the system.

**FIGURE 3:**
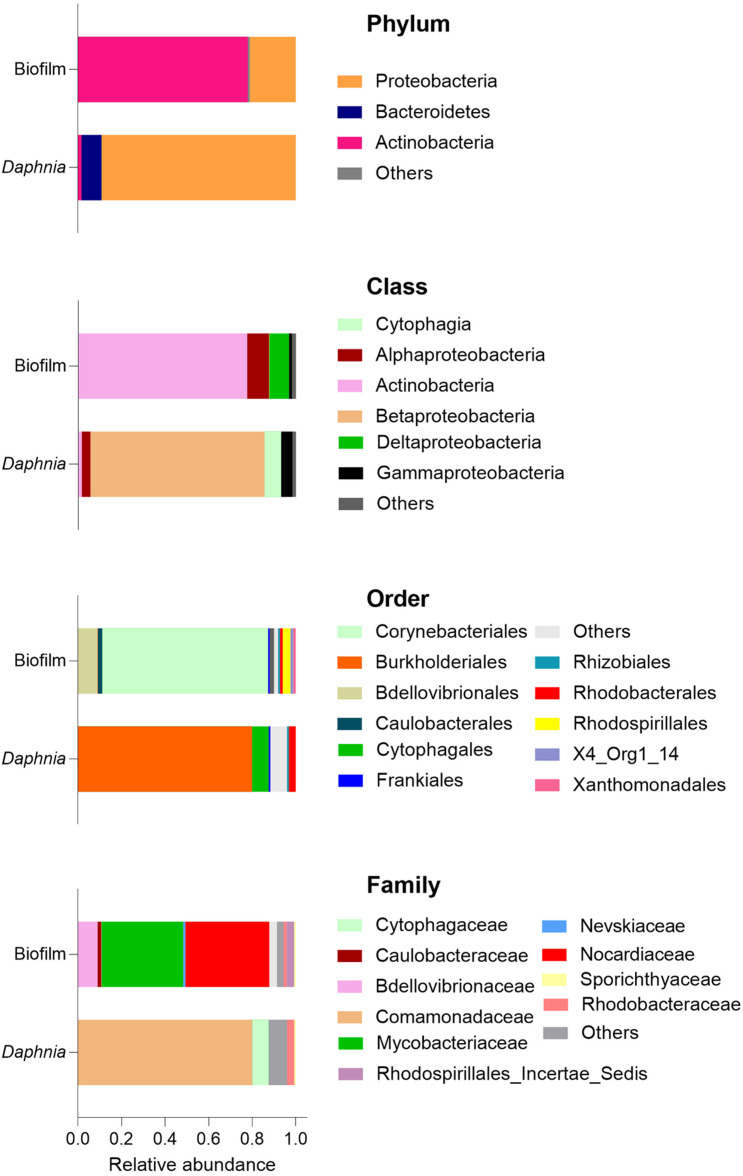
Dominance structure of the biofilm (*n* = 15) and *Daphnia* microbiome (*n* = 3) communities. The relative contributions of the taxa were averaged for each sample type. See also Supplementary Figures 3, 4.

**FIGURE 4:**
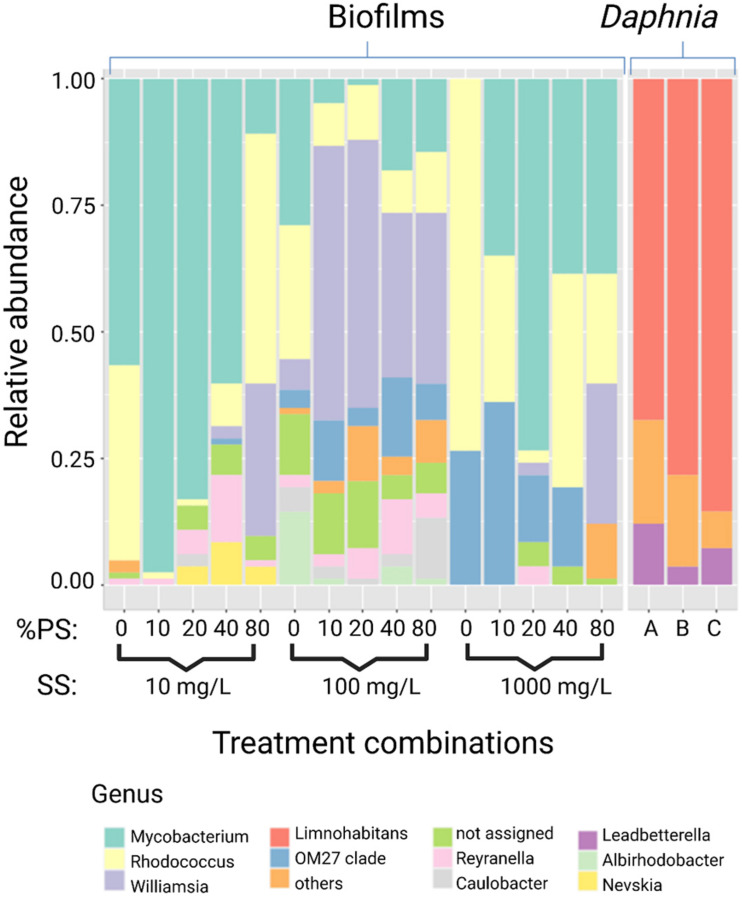
Relative abundance of the most abundant genera in the biofilms recovered from the particulate matter in the assay (*n* = 15) and *Daphnia magna* microbiome (*n* = 3). See Figure 3 for the overview of the higher taxonomic levels.

Regarding the taxonomic characterization, there were no common dominant taxa at any taxonomic level for the biofilm and the microbiota samples (Figures 3, 4 and Supplementary Figure 3). The *Daphnia* microbiota was dominated by Comamonadaceae (Proteobacteria; Figure 3), with the most common genera *Limnohabitans* and *Leadbeterella* (Figure 4). In the biofilm communities associated with particulate matter, the dominating phyla were Actinobacteria, represented by class Corynebacteriales, and Deltaproteobacteria represented by class Bdellovibrionales (Figure 3). The Corynebacteriales were represented by two families, Nocardiaceae, with genera *Williamsia* and *Rhodococcus*, and Mycobacteriaceae, with its only known genus *Mycobacterium* (Figure 4). Family Bdellovibrionaceae represented class Bdellovibrionales, with the OM27 clade as a single dominant member. Thus, the communities were dominated by four genera, three of which are Gram-positive Actinobacteria (*Mycobacterium*, *Williamsia*, and *Rhodococcus*), and one is a Gram-negative deltaproeobacterium (OM27 clade). Of Proteobacteria, the genus *Reyranella* was the most abundant; *Caulobacter* and *Nevskia* were also present, albeit at low abundances (Figure 4). Accordingly, Actinobacteria and Deltaproteobacteria represent the core microbiome associated with particulate matter, representing >90% of the ASVs assigned at the phylum level (Supplementary Figure 4).

**FIGURE 5:**
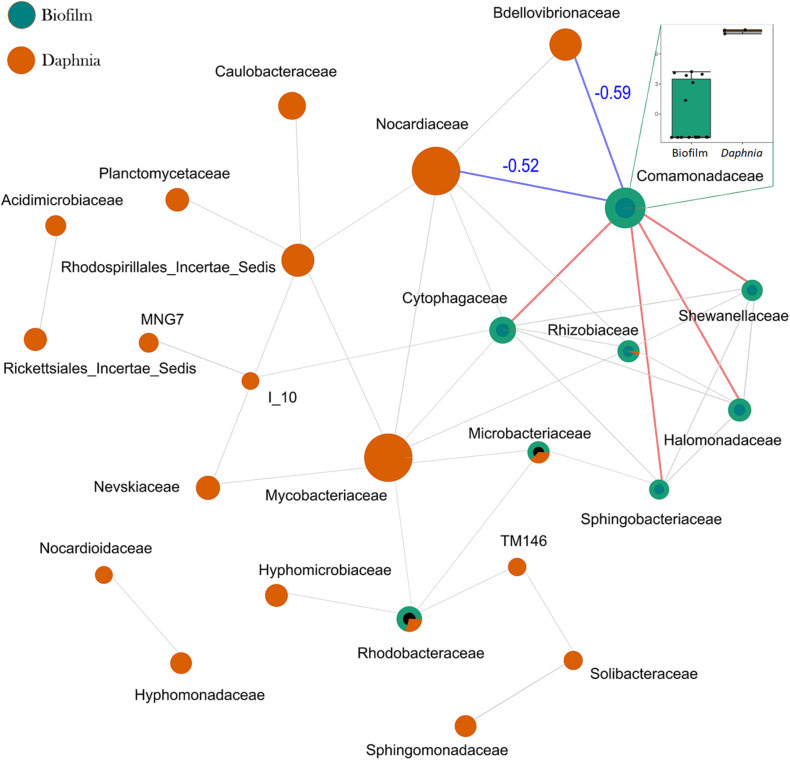
SprCC correlation network for communities of biofilm and *Daphnia* microbiota. The nodes represent taxonomic features (families in this case; the node size is based on the number of connections), and edges represent correlations greater than the correlation threshold of 0.3 with *p* < 0.05 between pairs of taxa. The line color reflects direction (red: positive; blue: negative) for the correlations that were found significantly influential by GLM; all other correlations are in gray. The correlation coefficients for the negative correlations are also shown in blue. The box plot on the insert compares the log-abundances of Comamonadaceae between the biofilm and *Daphnia*. A complete list of significant SparCC correlations is provided in Supplementary Table 4.

SparCC analysis conducted with samples stratified according to the origin (biofilm and *Daphnia*) examined the relationships across taxa in each sample type. The co-exclusion relationship between the most common members of *Daphnia* microbiota (Comamonadaceae) and the dominant biofilm bacteria Nocardiaceae and Bdellovibrionaceae as well as between Cytophagaceae/Rhizobiaceae and Mycobacteriaceae were found (Figure 5 and Supplementary Table 4).

### Effects of Experimental Factors and Biofilms on *Daphnia* Mortality

**FIGURE 6:**
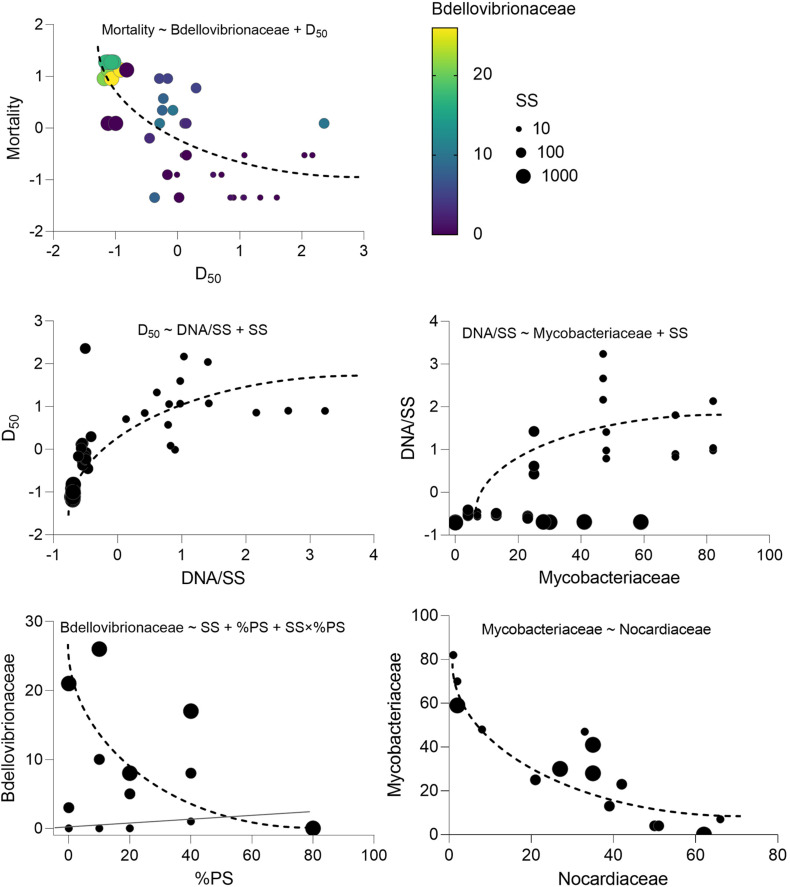
Key variables behind the observed *Daphnia* mortality variation in the experiment and the relationships driving their variability. The model indicated on each panel shows the predictors for each dependent variable that directly or indirectly influences. Mortality and corresponds to the regressions with significant effects identified in the best-fit models; see Table 2 for the GLM output and Supplementary Table 3 for the full list of the potential predictors tested.

**FIGURE 7:**
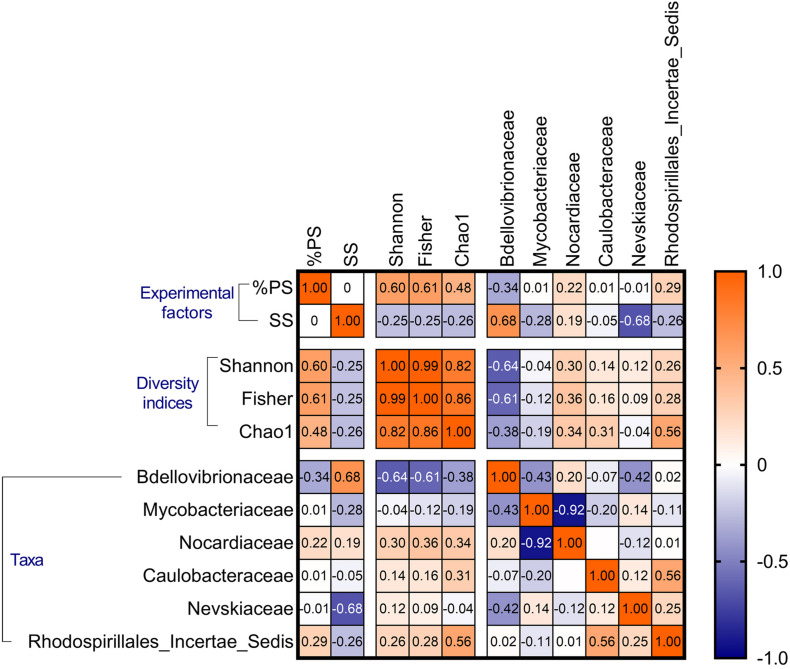
Spearman’s cross-correlations (*rho* values) for the biofilm diversity indices (Shannon–Wiener, Fisher’s alpha, and Chao1) and the abundance of the most common families. The experimental factors (SS and %PS) are also included. See Supplementary Table 5, for the *p*-values of these correlations.

There was no daphnid mortality in the controls, whereas exposure to the test mixtures resulted in the mean mortality values varying from 6 to 86% in the lowest and highest SS levels, respectively (Supplementary Figure 5). The best-fit model for mortality identified the median aggregate size and Bdellovibrionaceae as the main drivers, with higher mortality observed in the mixtures with small aggregates and high Bdellovibrionaceae contribution (Table 2 and Figure 6). These drivers were, in turn, positively affected by SS concentration (Table 2), which was behind the dose-response mortality (Supplementary Figures 5, 6). Moreover, there were significant positive effects of DNA/SS and %PS on D_50_ and Bdellovibrionaceae, respectively, with concomitant alleviation of the adverse effects. The DNA/SS values were also stimulated by Mycobacteriaceae and decreased at higher SS levels (Figure 6). Notably, the DNA amount in the test system was not related to *Daphnia* mortality, which supports the use of DNA/SS ratio as a proxy for biofilm thickness. Finally, the single best predictor of Mycobacteriaceae was Nocardiaceae relative abundance (Table 2 and Figures 6, 7). Overall, pathways related to the %PS and well-developed biofilms rich in Mycobacteriaceae contribute to the lower daphnid mortality, whereas high SS and Bdellovibrionaceae abundance were associated with high mortality.

**TABLE 2:** Best-fit GLM models for *Daphnia* mortality, aggregate size (D_50_), biofilm thickness (DNA/SS), total DNA amount in the system (DNA), and influential taxonomic groups.

**Response variable**	**Best-fit predictors**	**Link**	**Estimate**	**Standard error**	**Wald**	***p***
Mortality	D_50_	Identity	–0.173	0.040	18.496	<0.0001
	Bdellovibrionaceae		0.021	0.005	19.729	<0.0001
D_50_	SS	Identity	–1.452	0.235	38.009	<0.0001
	DNA/SS		0.833	0.236	12.525	0.0004
Bdellovibrionaceae	SS	Log	5.729	0.604	90.046	<0.0001
	%PS		–3.123	0.597	26.755	<0.0001
	%PS × SS		–3.242	0.610	28.203	<0.0001
DNA/SS	SS	Identity	–0.004	0.0008	22.735	<0.0001
	Mycobacteriaceae		0.0001	0.00003	9.971	0.0016
DNA	SS	Log	0.158	0.040	15.377	<0.0001
Mycobacteriaceae	Nocardiaceae	Log	–3.159	0.695	20.682	<0.0001

*Meaningful interaction effects were first included in all models but omitted if found non-significant.*

*Mortality and abundance values were Box–Cox transformed, and all predictors were transformed to their *z* scores and centered to enable interpretation of the main effects when interaction terms were significant.*

### Bacterial Interactions in the Biofilm Communities Under Exposure to Suspended Solids

There were significant positive %PS effects on all bacterial diversity indices, whereas SS effects were negative, weaker, and significant only for Chao1 (Table 3 and Supplementary Figure 7). These responses to the experimental factors underlined the similarity in the alpha diversity responses across the dataset (Figure 7). The aggregate size variance (defined as Sorting; Supplementary Text 1 and Supplementary Table 1) was also a significant negative predictor for Fisher’s alpha and Chao1 (Table 3), indicating lower alpha diversity in the mixtures with more heterogeneous PSD. Finally, Bdellovibriaceae was a strong significant negative predictor for Shannon–Wiener and Fisher’s alpha indices (Table 3, Figure 7 and Supplementary Figure 7), and this variable was also present in 8 of the 10 top models for Chao1 (data not shown).

**TABLE 3:** GLM results for the diversity indices in the biofilm communities as a function of the experimental factors (SS and %PS) and relative abundance of the dominant taxa.

**Diversity index**	**Best-fit predictors**	**Link**	**Estimate**	**Standard error**	**Wald**	***p***
Shannon–Wiener	%PS	Identity	0.006	0.003	5.696	0.017
	Bdellovibrionaceae		–0.079	0.009	76.695	<0.0001
Fisher’s alpha	%PS	Identity	14.486	2.972	23.756	<0.0001
	Sorting		–7.464	2.808	7.068	0.008
	Bdellovibrionaceae		–1.984	0.364	29.733	<0.0001
Chao1	%PS	Log	22.947	4.571	25.200	<0.0001
	SS		20.795	4.606	20.380	<0.0001
	Sorting		–10.750	4.607	5.445	0.019

*The %PS × SS interaction term was first included in all models but omitted if found non-significant.*

*Abundance values were Box–Cox transformed, and all predictors were transformed to their *z* scores and centered to enable interpretation of main effects when the interaction term was significant.*

*See Figure 6 for visualization of the models.*

*See Supplementary Figure 9 for graphical presentation of the data.*

**FIGURE 8:**
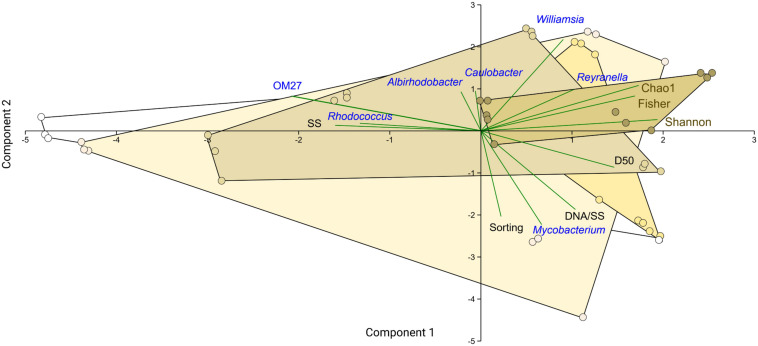
PCA biplot showing ordination based on the relative abundance of the common bacterial genera in the biofilm (blue), particle suspension concentration and size distribution parameters (SS, D_50_, and Sorting; black), and diversity indices (Chao1, Shannon, and Fisher; brown). The samples are grouped by %PS that is color-coded by the convex hull, with color intensity increasing from 0 to 80%. The first two components explain 58% of the variability in the dataset.

Principal component analysis showed the biofilms change along the %PS gradient, with an increase in the relative abundance of genera *Williamsia* (Nocardiaceae) and *Reyranella* (Rhodospirillales Incertae Sedis) at increasing polystyrene levels in the mixtures (Figure 8). These were also the genera associated with the high biofilm diversity, as seen from their aligning with the diversity indices. The high contribution of biofilms to the suspended matter and high variance of the aggregate size (Sorting) were associated with *Mycobacterium* (Mycobacteriaceae) contribution to the community (Figure 8). Finally, the high levels of the suspended matter (SS) and the prevalence of small-sized particles were beneficial for the OM27 clade (Bdellovibrionaceae) and *Rhodococcus* (Nocardiaceae); the increase in these genera was also related to the decrease in the diversity indices (Supplementary Table 5 and Figure 8).

## Discussion

### *Daphnia* Microbiota Is a Source of Biofilms in the Test System

In ecotoxicity assay using no-food exposure to suspended particulates, we found that *Daphnia* microbiota served as an inoculum for biofilm formation in the study system. Moreover, the relative polystyrene contribution to the particle mixture and the amount of the suspended solids were significant selective factors for the biofilm communities, which, in turn, affected particle aggregation and animal survival. Thus, the hypothesized ability of a test animal’ microbiota to colonize available surfaces in the exposure system impinging on the test outcome was confirmed. Although we demonstrated the microbiota transfer from the host to the biofilms using a single *D. magna* clone, these findings are generalizable for any ecotoxicity assay in animal testing. However, due to the inter-clonal variability in *Daphnia* microbiota, animal behavior, and environmental factors (Sullam et al., 2018; Mushegian et al., 2019), various patterns in the composition of the biofilm-associated microorganisms may be observed.

The biofilm community structure was remarkably different from that in the *Daphnia* microbiome: taken together, the taxa that were important in the biofilms contributed less than 1% in the inoculum. The *Daphnia* microbiome was dominated by Proteobacteria and Bacteroidetes, which is in line with other reports (Qi et al., 2009; Eckert et al., 2016; Callens et al., 2018; Mushegian et al., 2018; Cooper and Cressler, 2020; Motiei et al., 2020) and was also found for our clone and culture conditions (Motiei et al., 2020). However, these taxa contributed less than 25% to the biofilms. Instead, Actinobacteria (∼75%), represented by family Nocardiaceae (38%; genera *Williamsia* and *Rhodococcus*) and Mycobacteriaceae (37%) represented by its single genus *Mycobacterium* dominated biofilm communities recovered from all particle mixtures (Figures 3, 4).

In freshwater environments worldwide, Actinobacteria are ubiquitous and highly abundant (Newton et al., 2011; Samad et al., 2020). However, Actinobacteria never dominate *Daphnia* microbiome, although Nocardioidaceae and Microbacteriaceae are often present (Qi et al., 2009; Eckert et al., 2016; Callens et al., 2018; Mushegian et al., 2018; Cooper and Cressler, 2020; Motiei et al., 2020). *In situ*, the microbiota of freshwater cladocerans and copepods have much lower Actinobacteria contribution than the ambient bacterioplankton (Samad et al., 2020); however, the mechanisms supporting this niche differentiation are not well understood. Many Actinobacteria have a high capacity toward producing cellulases and antimicrobial substances, which predisposes them to engage in defensive symbioses with various herbivorous arthropods (Salem et al., 2013). They are also essential degraders of plant material, and some, e.g., *Rhodococcus* present in our biofilms (Figure 4), are known to degrade chitin, cellulose, and a broad range of other organic compounds (Jacquiod et al., 2013). However, high nutrient levels generally select against Actinobacteria (Haukka et al., 2006), which may disfavor these bacteria in digestive tracts naturally rich in nutrients compared to the surrounding water or suspended solids as in our experiment. Moreover, Actinobacteria are resistant to grazing by protozooplankton because of the S-layer in the bacterial cell wall (Tarao et al., 2009); although it is unclear whether this feature may affect bacteria intake by mesozooplankton, such as daphnids. In a grazing experiment with metacommunities (Berga et al., 2015), the relative abundance of Actinobacteria and Sphingobacteria increased in the presence of *D. magna*. The authors suggested that either the daphnids removed protozoans grazing on these bacteria or the latter were grazing-tolerant, supporting their spread in the bacterioplankton community. In our experiment with no protozoans present in the system, Actinobacteria released from the predation were the main group associated with particle aggregates, and, probably, the exposure media. In *Daphnia* culture, Actinobacteria were found to contribute much more to the culture media than to the *D. magna* microbiome (Cooper and Cressler, 2020). Hence, the differences in Actinobacteria between the biofilms and the host microbiota suggest that they are selected against by the animal microbiome, among other ecological factors.

The diversity was higher and appeared to be more variable in the biofilms than the inoculum (Figure 1 and Supplementary Figure 7), which was strongly related to the higher incidence of rare ASVs in the biofilms. A significant difference between the sample types was found for the Chao1 estimator (Figure 1), which is more sensitive to the rare taxa presence (Chao, 1984), but not for the other two indices. Notably, comparing the diversity indices between the biofilm and daphnid samples was partially hampered by the small sample size (three samples) for the *Daphnia* microbiota. Nevertheless, although the diversity and abundance of the key taxa in the gut are, at least to some extent, under control by the host animal, the microbiome source for planktonic crustaceans is the ambient bacterioplankton (Tang et al., 2010). Therefore, if suspended particulate matter provides niches for the enrichment and diversification of the rare and potentially harmful taxa, their intake and contribution to the animal microbiome may increase, both in the experimental and, possibly, *in situ*.

### Selection of Biofilm Components by Suspended Solids

As hypothesized, mixture composition (i.e., the relative PS contribution) and the total amount of suspended solids favored preferential growth of rare members within the daphnid microflora. Genera *Williamsia*, *Mycobacterium* (both Actinobacteria), and *Reyranella* (Proteobacteria) were the key members of the core biofilm community. Moreover, *Williamsia* and *Reyranella* were associated with the high %PS and high biofilm diversity (Figures 4, 7). These genera of Actinobacteria are known hydrocarbon degraders (Yassin et al., 2007; Bell et al., 2016), which points toward the possibility of utilizing plastic as a carbon source and a selection for organotrophic taxa present in the *Daphnia* microbiome.

In line with our previous findings (Ogonowski et al., 2018; McGivney et al., 2020), high contribution of polystyrene (%PS) significantly stimulated biofilm diversity (Figure 7), whereas high Bdellovibrionaceae contribution decreased it (Supplementary Figure 7). *Bdellovibrio* and like organisms (BALOs) are a monophyletic group of Gram-negative obligate prokaryotic predators, ubiquitous in the aquatic environment and natural biofilms (Williams et al., 1995; Rotem et al., 2014). BALOs display niche separation, different predation strategies, and prey selectivity such that some BALOs are more specific for particular prey organisms, while others are more prey generic (Rogosky et al., 2006). Bdellovibrionales comprises families Bdellovibrionaceae, which belongs to δ-proteobacteria, and the genus *Micavibrio*, which belongs to α-proteobacteria (Rotem et al., 2014; Koval et al., 2015). In our dataset, the Bdellovibrionaceae dynamics were driven by the OM27 clade, a cluster of unculturable bacteria phylogenetically related to *Bdellovibrio* (Orsi et al., 2016), the type genus preying on Gram-negative bacteria by burrowing into and occupying the periplasmic space between the inner and outer membranes of the prey. The predation on various Gram-negative bacteria originated from the *Daphnia* microbiome was the most likely reason for the decreasing biofilm diversity with an increase of the OM27 clade across the treatments.

**FIGURE 9:**
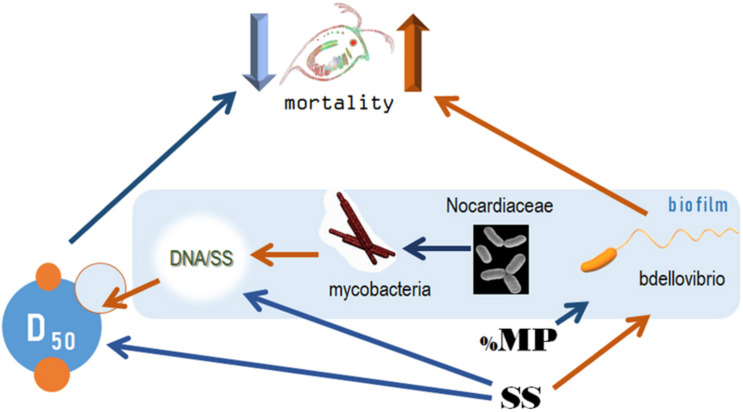
The pathways for the causal relations behind the variability in the mortality rate under the experimental exposure conditions. Orange and blue arrows depict positive and negative relationships, respectively. SS, concentration of suspended solids (mg/L); %PS, proportion of polystyrene in the exposure mixture; D_50_, median aggregate size (μm); DNA/SS, relative contribution of the biofilm to the particle mixture (μm/mg). The bacteria taxa are the dominant families in the communities, see Figure 3 and Supplementary Figure 3.

As Bdellovibrios are surface-associated organisms (Williams et al., 1995), the suspended solid concentration facilitated their abundance in biofilms, whereas the increased polystyrene proportion was a negative predictor (Table 2). Therefore, both the total colonizable surface area and the physicochemical properties of this surface are significant drivers of the propagation of these taxa in the test system and, possibly, in the environment. It is also plausible that these experimental factors were decisive for the non-BALOs biofilm formation, with the bottom-up effects on the OM27 success. Notably, under *Bdellovibrio* predation in metacommunities, the Gram-positive bacteria *Mycobacterium*, *Williamsia*, and *Rhodococcus*, were found to benefit from the decline of the Gram-negative members (Dashiff et al., 2011). Therefore, it is possible that in our experiment, *Mycobacterium* was growing well in the biofilms under the predation pressure by OM27 bacteria, which contributed to the biofilm thickness (Figure 9). The controlling factor for the *Mycobacterium* abundance was primarily Nocardiaceae (Table 2), indicating that biotic interactions in the biofilms were crucial, both for the biofilm development and the downstream effects on particle aggregation and behavior (Figure 9).

### Biofilms and Particle Aggregation Are the Main Drivers of *Daphnia* Mortality

Daphnid survival in the experiment was affected by particle aggregation and biofilm composition, namely Bdellovibrionaceae relative abundance (Figure 9). The high death rate was associated with a smaller aggregate size and high Bdellovibrionaceae contribution to the biofilm communities. The adverse effects of suspended solids on the test animals were mediated by the aggregate size (high SS → smaller particles → higher mortality) and relative abundance of Bdellovibrionaceae (high SS → more predatory bacteria → higher mortality). The effect of polystyrene contribution to the suspension was ameliorating and also mediated by its negative impact on Bdellovibrionaceae (Figures 6, 9).

Whereas the particle size as a mediator has been described in other tests with suspended solids (Kirk, 1991; Rehse et al., 2016; Mattsson et al., 2018), the biofilm composition effects are less known. SparCC analysis indicated a negative correlation between Bdellovibrionaceae and Comamonadaceae, which constitute a core *Daphnia* microbiome (Figure 5), which may contribute to the adverse effect of Bdellovibrionaceae on animal survival. A plausible explanation is that Bdellovibrionaceae preyed on the Gram-negative Comamonadaceae and, possibly, other Gram-negative proteobacterial taxa resulting in a selective advantage for Actinobacteria (Gram-positive, not known to be prey for BALOs) and other rare species. These relations are in line with other observations that BALOs can drive microbiome alpha-diversity in different animal species and environments (Johnke et al., 2020). One can also speculate that overall biofilm diversity that was positively associated with %PS (Figure 7) was not beneficial for *Daphnia* survival due to the high contribution of rare and possibly harmful taxa, such as Nocardiaceae (Figure 6) that are rich in taxa with a saprophytic lifestyle and many members of this family are opportunistic pathogens (Goodfellow, 2014). The negative correlation between Nocardiaceae and Comamonadaceae (Figure 5) supports this explanation.

In conclusion, our findings suggest that bacteria originated from *Daphnia* microbiota established biofilm communities during the 4 days of the exposure. In these biofilms, rare, predatory, and potentially pathogenic taxa were overrepresented. The biofilm thickness and composition were influential for the daphnid mortality, acting via particle aggregation and, possibly, interactions with the animal microbiota (Figure 9). Contrary to the commonly assumed adverse effect of the microplastics, the polystyrene addition to the test mixture alleviated the hazard potential. These findings suggest that ecological and microbe–microbe interactions in bacterial biofilms developing in the ecotoxicity assays can favor potentially harmful taxa with concomitant effects on the test animals. In effect studies with solid waste materials and filter-feeders sensitive to PSD, these community-level interactions can also lead to particle aggregation changes, with indirect effects on the test animals.

## Data Availability Statement

The datasets presented in this study can be found in the online repository. The name of the repository and accession numbers can be found in the article/Supplementary Material.

## Author Contributions

EG and RE-S formulated the research question. AM conducted the experiment, analyzed PSD samples, processed the data, and prepared samples for DNA analysis. EG conducted the statistical analyses. RE-S carried out the interpretation of the results. EG compiled the first draft of the manuscript with contributions from RE-S. All authors contributed to the manuscript writing.

## Conflict of Interest

The authors declare that the research was conducted in the absence of any commercial or financial relationships that could be construed as a potential conflict of interest.
