# Supplementary material for: Understanding Biofilm Formation in Ecotoxicological Assays With Natural and Anthropogenic Particulates
Source: Front Microbiol. 2021 Jul 1;12:632947. doi: 10.3389/fmicb.2021.632947 (PMC8281255; doi:10.3389/fmicb.2021.632947)
Supplement: Supplementary file 1 [file Data_Sheet_1.docx]

Supplementary Material

# Supplementary Text S1: Particle suspension preparation and size distribution measurements

## Test microplastic and kaolin

Polystyrene (PS; Goodfellow GmbH, product number ST316311) was obtained as 3–5 mm-sized granules from the manufacturer and milled to a powder by Messer Group GmbH, Germany. The PS powder was mixed with Milli-Q water containing 0.01% v/v of a non-ionic surfactant (Tween-80, Sigma-Aldrich); the concentration of Tween-80 in the final exposure media was 0.001%, which is below toxicity level for *Daphnia*, bacteria and other microorganisms (Lechuga et al., 2016; Nielsen et al., 2016). When preparing the test mixtures, the stock suspensions were sonicated for 10 min before use in order to minimize particle aggregation. Using a metal Büchner funnel with a 0.2-µm nylon membrane (Merck Millipore, GNWP04700), the <40 µm fraction was obtained; the filtrate was dried, weighed**,** and re-suspended for size distribution analysis and use in the experiments.

Kaolin (Sigma-Aldrich, K7375), particles of laminar shape, contains mainly the clay mineral kaolinite, a hydrous aluminosilicate. It is present globally in suspended particulates and has been used as reference material when assessing microplastic effects (Gerdes et al. 2019) and as a test material when assessing effects of exposure to suspended solids (Gordon and Palmer 2015). Here, a measured dosage of kaolin was first added into the water of 0.5 L to prepare suspension with 10 g/L solid concentration. Then, the suspensions were mixed with an electronic stirrer for 30 min. After that, the suspensions were processed by ultrasonic disperser for 5 min to wet the particles and ensure full dispersion.

- 1. **Particle size measurements**

The particle size distribution (PSD) characterizes the relationship between particle abundance and size. Laser diffraction measurements are commonly used to measure PSD in suspended sediments because the measurement process is less disturbing to the aggregates than other methods (Serra et al. 2001). The measurement principle is based on the light scattering by passing a rotating laser beam through the glass wall and focusing the near-angle light pulses from the laser beam/particle collisions onto the photodetector; the collision rate is then converted into particle count and size data.

Laser particle counter (Spectrex, model PC-2000, Redwood City, USA) with integrated counting mode (32 bins) in the 1-100 µm range was used to measure PSD in all test mixtures. The instrument performance was verified daily using a standard (4.2 µm polystyrene spheres in an alcohol/freon liquid) and standard blank provided by the manufacturer and/or particle-free water. Testing replicate samples of the polymer and kaolin standards showed that the between-replicate variation of particle counts was less than 6 % for <10 µm range and less than 3 % for < 60 µm range.

GRADISTAT program, version 8.0 (Blott and Pye 2001) was used to analyze PSD data according to the method by Folk and Ward (Folk and Ward 1957). We calculated mean and median (D_50_) particle sizes, mode particle size for non-unimodal distributions, particle size which 10 % of the sample is below (D_10_), particle size which 90 % of the sample is above (D_90_), sample sorting (σ), skewness, and kurtosis.

# Supplementary Figures and Tables for PSD analysis in stock suspensions

**Supplementary Table S1.** Particle size distribution parameters calculated by GRADISTAT for stock suspensions of kaolin and PS. Three technical replicates were obtained for each spectrum and averaged.

| **Distribution parameters, μm** | **Material** | |
| --- | --- | --- |
|  | **Kaolin** | **Polystyrene** |
| Sample type | Unimodal | Unimodal |
| Mean | 9.6 | 8.7 |
| D_10_ | 7.602 | 9.645 |
| D_50_  (median) | 9.090 | 11.23 |
| D_90_ | 10.69 | 13.48 |
| D_90_ / D_10_ | 1.407 | 1.397 |
| D_90_ - D_10_ | 3.092 | 3.832 |
| Geometric mean (*x*) | 9.107 | 11.28 |
| Sorting (σ) | 1.136 | 1.246 |
| Skewness (*Sk*) | -0.032 | 0.350 |
| Kurtosis (*K*) | 0.886 | 2.524 |

# Supplementary Figures and Tables for bacteria community analysis

**Supplementary Figure S1.** Library size overview for samples of *Daphnia magna* (D1, D2, and D3) used to inoculate the system with bacteria and biofilms associated with the particulate matter in the exposure. The first number in the biofilm sample code indicates the suspended solid concentration (mg/L) and the number after the underscore is the %PS in the suspension, e.g., 1000_10 stands for 1000 mg/L suspended solids with 10% polystyrene. These data were used for filtering and normalization before the statistical analyses.


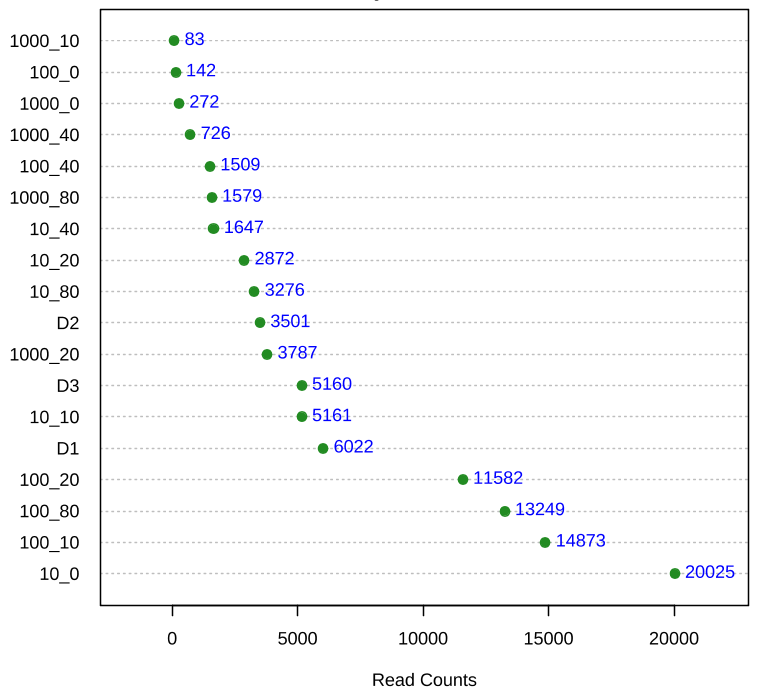


Sample code

# Supplementary Figure S2. Rarefaction curves showing the level of ASV saturation in the samples of (A) biofilms associated with the particulate matter in the exposure and (B) *Daphnia magna* used as an inoculum. Sample coding as in Figure S1. The inserts show samples rarefied to 83 ASV, which was the minimum library size in the dataset (Figure S1).

#
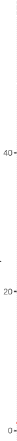

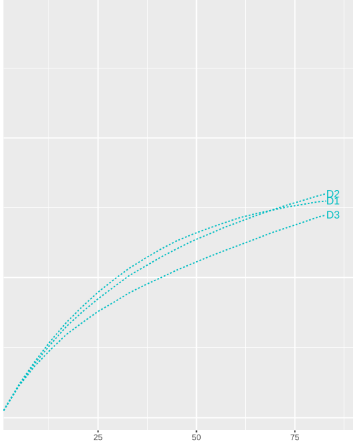

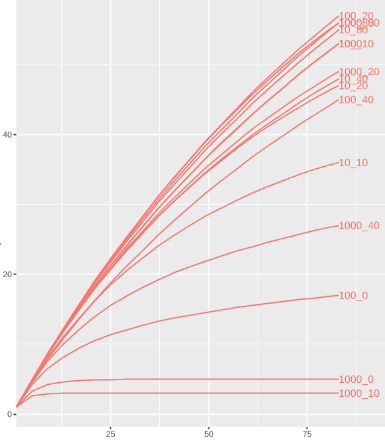


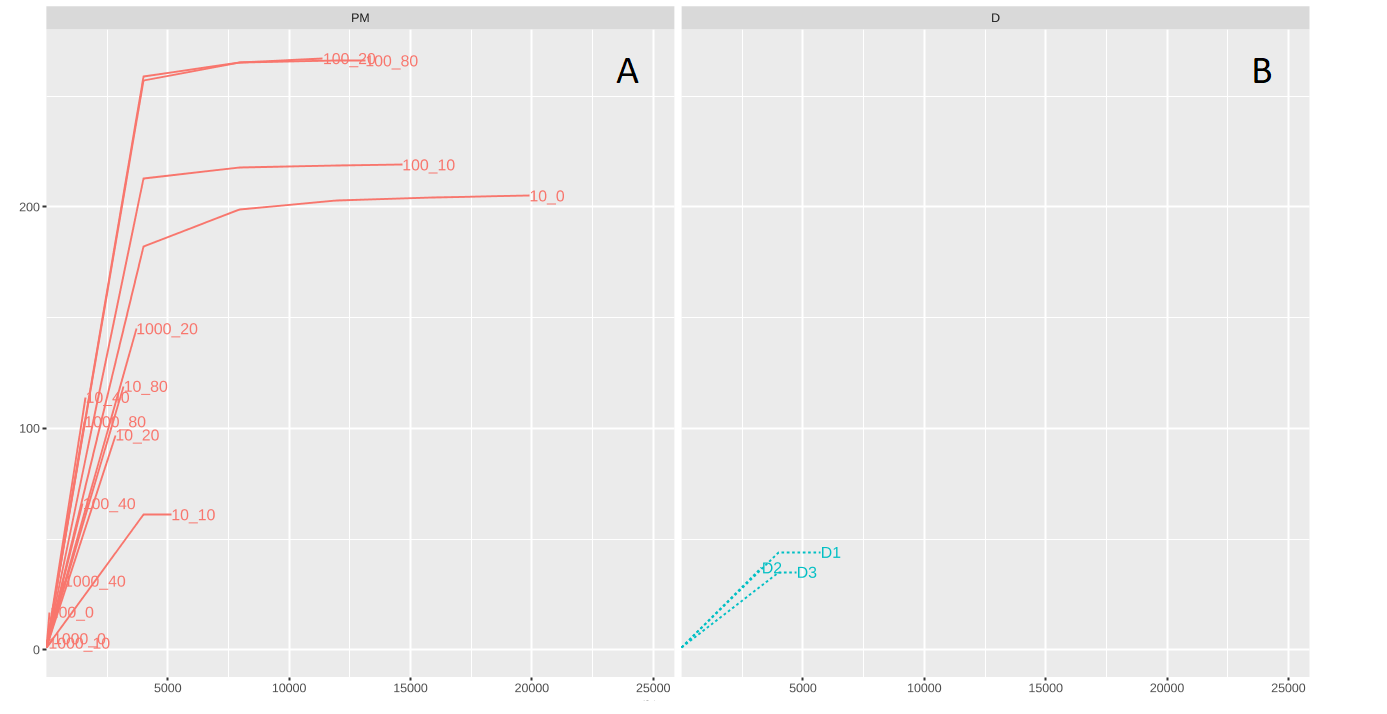


ASV richness

Sequence sample size

**Supplementary Table S2.** Unpaired t-test with Welch's correction for comparison of the diversity indices between the biofilm and *Daphnia* microbiota used as inoculum; see Figure 1. To compare variances between the groups, F test was used. Significant *p* values (two-tailed) are in bold face.

| **Diversity indices** | **Welch-corrected t** | ***p* value** | **F test**  F, DFn, Dfd | ***p* value** |
| --- | --- | --- | --- | --- |
| Chao1 | t=2.345, df=12.85 | **0.035** | 12.32, 14, 2 | 0.155 |
| Simpson | t=1.854, df=3.419 | 0.149 | 1.584, 14, 2 | 0.907 |
| Shannon-Wiener | t=1.599, df=3.811 | 0.188 | 1.979, 14, 2 | 0.772 |

**Supplementary Figure S3.** Community composition at the family level of (A) biofilms recovered from the particulate matter in the assay (*n* = 15) and (B) *Daphnia magna* microbiome (*n* = 3). Mean values for each group were used to calculate the contributions.


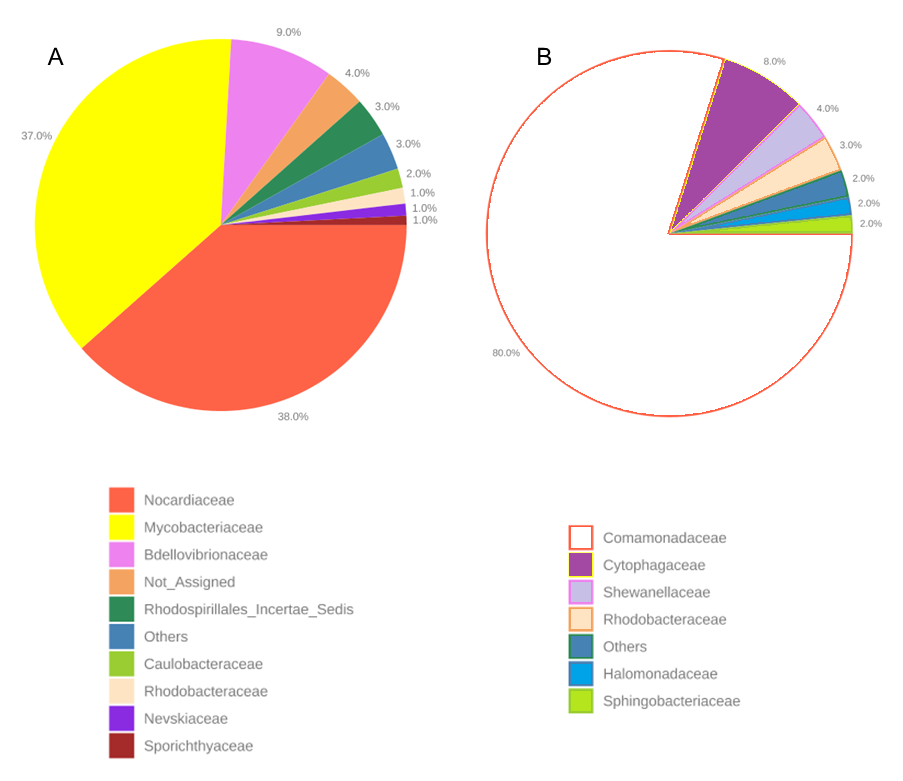


**Supplementary Figure S4.** The core microbiome defined by both prevalence and relative abundance at the family for (A) *Daphnia magna* used as an inoculum (*n* = 3), and (B) biofilms associated with the particulate matter in the exposure (*n* = 15). To detect the core microbiome, 20% prevalence and 0.01% relative abundance thresholds were used.

*Daphnia magna*


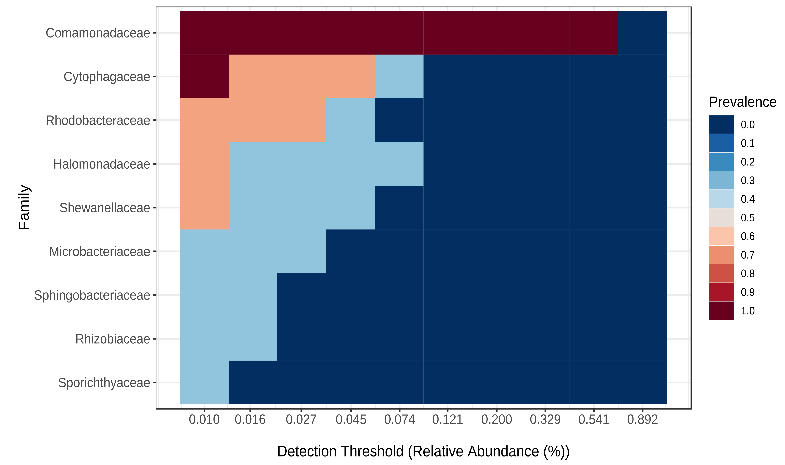

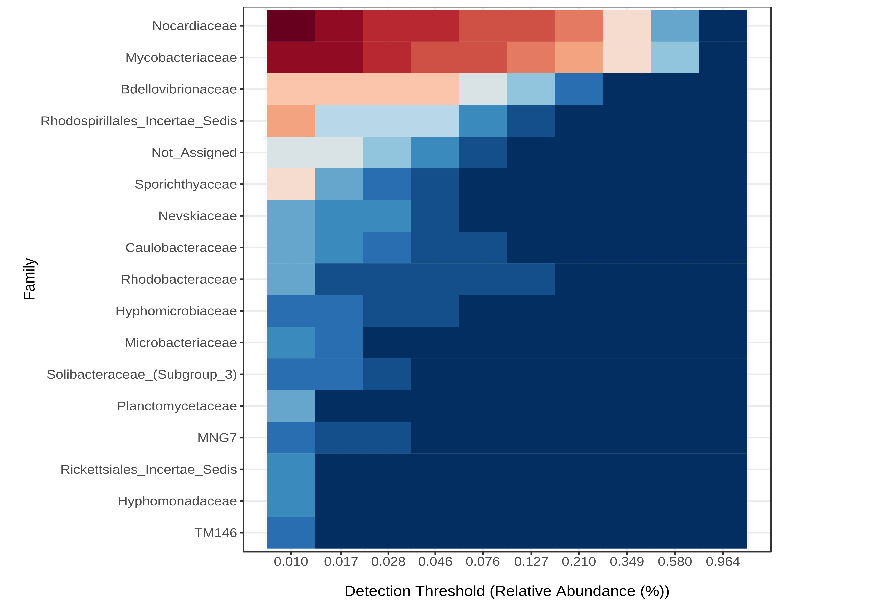


Families

A

B

Biofilms

**Supplementary Table S3.** Design of GLMs for different response variables starting with the model identifying significant predictors of mortality, and then sequentially evaluating variables driving variability of these predictors. The experimental factors (SS and %PS as well as their interaction) were included in each model. Predictors used in the best-fit models are in bold face.

| **Response variable** | **Tested predictors** |
| --- | --- |
| Mortality | SS, %PS, **D_50_**, SD, DNA, DNA/SS, **Bdellovibrionaceae**, Nocardiaceae, Mycobacteriaceae |
| D_50_ | **SS**, %PS, DNA, **DNA/SS** |
| Bdellovibrionaceae | **SS, %PS,** D_50_, DNA, DNA/SS, Nocardiaceae, Mycobacteriaceae |
| DNA/SS | **SS**, %PS, D_50_, Bdellovibrionaceae, Nocardiaceae, **Mycobacteriaceae** |
| Mycobacteriaceae | SS, %PS, **Nocardiaceae**, Bdellovibrionaceae |
| DNA* | **SS**, %PS, Mortality, Bdellovibrionaceae, Nocardiaceae, Mycobacteriaceae |

*DNA was not identified as a significant predictor in any path leading to the alterations in *Daphnia* mortality; here, we used GLM analysis to identify whether presence of dead daphnids can increase amount of free DNA in the system and thus affect interpretation of DNA/SS as a proxy for relative contribution of biofilm into particulate matter in the test suspensions, i.e., biofilm thickness.

**Supplementary Table S4.** A complete list of significant SparCC correlations based on the dataset that included both the biofilm associated with the particulate material and *Daphnia magna* microbiota; the analysis was conducted at family level. See Figure 3 for the network diagram.

| **Taxon1** | **Taxon2** | **Correlation** | ***p*** |
| --- | --- | --- | --- |
| Acidimicrobiaceae | Rickettsiales_Incertae_Sedis | 0.759 | 0.0099 |
| Bdellovibrionaceae | Comamonadaceae | -0.5869 | 0.0198 |
| Bdellovibrionaceae | Nocardiaceae | 0.6288 | 0.0198 |
| Caulobacteraceae | Not_Assigned | 0.5315 | 0.0099 |
| Caulobacteraceae | Rhodospirillales_Incertae_Sedis | 0.6031 | 0.0396 |
| Comamonadaceae | Bdellovibrionaceae | -0.5869 | 0.0198 |
| Comamonadaceae | Cytophagaceae | 0.7839 | 0.0099 |
| Comamonadaceae | Halomonadaceae | 0.7018 | 0.0099 |
| Comamonadaceae | Nocardiaceae | -0.5241 | 0.0198 |
| Comamonadaceae | Shewanellaceae | 0.6912 | 0.0099 |
| Comamonadaceae | Sphingobacteriaceae | 0.5569 | 0.0198 |
| Cytophagaceae | Comamonadaceae | 0.7839 | 0.0099 |
| Cytophagaceae | Halomonadaceae | 0.8274 | 0.0099 |
| Cytophagaceae | I_10 | 0.4742 | 0.0495 |
| Cytophagaceae | Mycobacteriaceae | -0.4479 | 0.0495 |
| Cytophagaceae | Nocardiaceae | -0.5357 | 0.0198 |
| Cytophagaceae | Rhizobiaceae | 0.6402 | 0.0099 |
| Cytophagaceae | Shewanellaceae | 0.8277 | 0.0099 |
| Cytophagaceae | Sphingobacteriaceae | 0.6897 | 0.0099 |
| Halomonadaceae | Comamonadaceae | 0.7018 | 0.0099 |
| Halomonadaceae | Cytophagaceae | 0.8274 | 0.0099 |
| Halomonadaceae | Rhizobiaceae | 0.6205 | 0.0297 |
| Halomonadaceae | Shewanellaceae | 0.9803 | 0.0099 |
| Halomonadaceae | Sphingobacteriaceae | 0.84 | 0.0198 |
| Hyphomicrobiaceae | Rhodobacteraceae | -0.4927 | 0.0495 |
| Hyphomonadaceae | Nocardioidaceae | -0.4561 | 0.0396 |
| I_10 | Cytophagaceae | 0.4742 | 0.0495 |
| I_10 | MNG7 | 0.798 | 0.0396 |
| Microbacteriaceae | Nevskiaceae | -0.5138 | 0.0297 |
| Microbacteriaceae | Not_Assigned | -0.5361 | 0.0198 |
| Microbacteriaceae | Rhodobacteraceae | 0.4529 | 0.0297 |
| Microbacteriaceae | Sphingobacteriaceae | 0.4549 | 0.0396 |
| MNG7 | I_10 | 0.798 | 0.0396 |
| Mycobacteriaceae | Cytophagaceae | -0.4479 | 0.0495 |
| Mycobacteriaceae | Nocardiaceae | -0.7671 | 0.0099 |
| Mycobacteriaceae | Not_Assigned | 0.6925 | 0.0099 |
| Mycobacteriaceae | Rhizobiaceae | -0.5517 | 0.0198 |
| Mycobacteriaceae | Rhodobacteraceae | -0.4109 | 0.0396 |
| Mycobacteriaceae | Rhodospirillales_Incertae_Sedis | 0.6009 | 0.0198 |
| Nevskiaceae | Microbacteriaceae | -0.5138 | 0.0297 |
| **Supplementary Table S4.** Cont. | | | |
| Nevskiaceae | Not_Assigned | 0.6103 | 0.0198 |
| Nevskiaceae | Rhodospirillales_Incertae_Sedis | 0.6052 | 0.0198 |
| Nocardiaceae | Bdellovibrionaceae | 0.6288 | 0.0198 |
| Nocardiaceae | Comamonadaceae | -0.5241 | 0.0198 |
| Nocardiaceae | Cytophagaceae | -0.5357 | 0.0198 |
| Nocardiaceae | Mycobacteriaceae | 0.7671 | 0.0099 |
| Nocardiaceae | Not_Assigned | 0.5202 | 0.0198 |
| Nocardiaceae | Rhizobiaceae | -0.5735 | 0.0099 |
| Nocardiaceae | Rhodospirillales_Incertae_Sedis | 0.4327 | 0.0495 |
| Nocardioidaceae | Hyphomonadaceae | -0.4561 | 0.0396 |
| Not_Assigned | Caulobacteraceae | 0.5315 | 0.0099 |
| Not_Assigned | Microbacteriaceae | -0.5361 | 0.0198 |
| Not_Assigned | Mycobacteriaceae | 0.6925 | 0.0099 |
| Not_Assigned | Nevskiaceae | 0.6103 | 0.0198 |
| Not_Assigned | Nocardiaceae | 0.5202 | 0.0198 |
| Not_Assigned | Rhodospirillales_Incertae_Sedis | 0.8276 | 0.0099 |
| Planctomycetaceae | Rhodospirillales_Incertae_Sedis | 0.5235 | 0.0297 |
| Rhizobiaceae | Cytophagaceae | 0.6402 | 0.0099 |
| Rhizobiaceae | Halomonadaceae | 0.6205 | 0.0297 |
| Rhizobiaceae | Mycobacteriaceae | -0.5517 | 0.0198 |
| Rhizobiaceae | Nocardiaceae | -0.5735 | 0.0099 |
| Rhizobiaceae | Shewanellaceae | 0.6264 | 0.0198 |
| Rhodobacteraceae | Hyphomicrobiaceae | -0.4927 | 0.0495 |
| Rhodobacteraceae | Microbacteriaceae | 0.4529 | 0.0297 |
| Rhodobacteraceae | Mycobacteriaceae | -0.4109 | 0.0396 |
| Rhodobacteraceae | TM146 | -0.4927 | 0.0099 |
| Rhodospirillales_Incertae_Sedis | Caulobacteraceae | 0.6031 | 0.0396 |
| Rhodospirillales_Incertae_Sedis | Mycobacteriaceae | 0.6009 | 0.0198 |
| Rhodospirillales_Incertae_Sedis | Nevskiaceae | 0.6052 | 0.0198 |
| Rhodospirillales_Incertae_Sedis | Nocardiaceae | 0.4327 | 0.0495 |
| Rhodospirillales_Incertae_Sedis | Not_Assigned | 0.8276 | 0.0099 |
| Rhodospirillales_Incertae_Sedis | Planctomycetaceae | 0.5235 | 0.0297 |
| Rickettsiales_Incertae_Sedis | Acidimicrobiaceae | 0.759 | 0.0099 |
| Shewanellaceae | Comamonadaceae | 0.6912 | 0.0099 |
| Shewanellaceae | Cytophagaceae | 0.8277 | 0.0099 |
| Shewanellaceae | Halomonadaceae | 0.9803 | 0.0099 |
| Shewanellaceae | Rhizobiaceae | 0.6264 | 0.0198 |
| Shewanellaceae | Sphingobacteriaceae | 0.8477 | 0.0099 |
| Solibacteraceae_(Subgroup_3) | Sphingomonadaceae | 0.8708 | 0.0198 |
| Solibacteraceae_(Subgroup_3) | TM146 | -0.4775 | 0.0396 |
| Sphingobacteriaceae | Comamonadaceae | 0.5569 | 0.0198 |
| Sphingobacteriaceae | Cytophagaceae | 0.6897 | 0.0099 |
| Sphingobacteriaceae | Halomonadaceae | 0.84 | 0.0198 |
| **Supplementary Table S4.** Cont. | | | |
| Sphingobacteriaceae | Microbacteriaceae | 0.4549 | 0.0396 |
| Sphingobacteriaceae | Shewanellaceae | 0.8477 | 0.0099 |
| Sphingomonadaceae | Solibacteraceae_(Subgroup_3) | 0.8708 | 0.0198 |
| TM146 | Rhodobacteraceae | -0.4927 | 0.0099 |
| TM146 | Solibacteraceae_(Subgroup_3) | -0.4775 | 0.0396 |

# Supplementary Figures and Tables for ecotoxicity analysis

# Supplementary Figure S5. The dose-response curves for mortality in *Daphnia magna* exposed to kaolin-polystyrene mixtures at 0 (Control), 10, 100 and 1000 mg/L of the total suspended solids (SS), and different contribution of the polystyrene to the mixture (%PS: 0 to 80%).

#

%PS: 0%

%PS: 10%

%PS: 20%

%PS: 40%

%PS: 80%

Log SS concentration, mg/L Log SS concentration, mg/L

Mortality, proportion

Log

**Supplementary Figure S6.** The responses of (A) mortality, (B) biofilm thickness (DNA/SS, µm/mg), and (C) aggregate size as D_50_ (µm) to the experimental treatment factors, proportion of polystyrene in the test mixtures (%PS) and concentration of suspended solids (SS, mg/L). The data point represents an average of three replicates, except for mortality, where the number of the observations was seven.


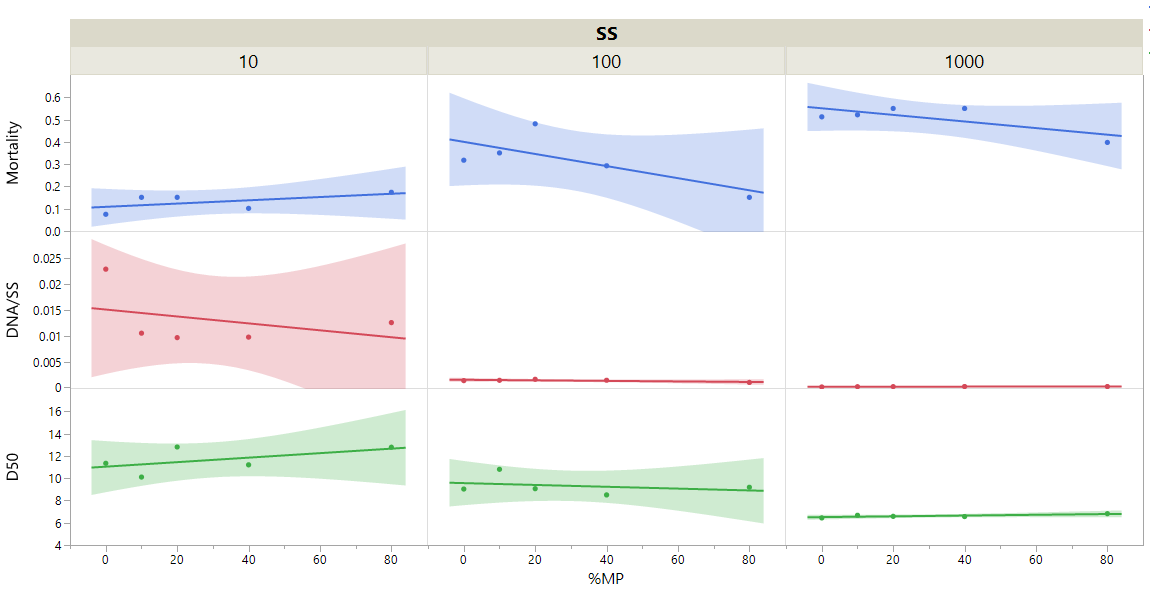


**(A)**

**(B)**

**(C)**

%PS

**Supplementary Table S5.** Estimated *p* values of the Spearman’s cross-correlations for the treatment factors (%PS and SS), biofilm diversity indices (Shannon-Wiener, Fisher’s alpha, and Chao1) and the relative abundance of common taxa. See Figure 5 for the *rho* values.

|  | %PS | SS | Shannon | Fisher's  alpha | Chao | Bdellovibrionaceae | Mycobacteriaceae | Nocardiaceae | Caulobacteraceae |
| --- | --- | --- | --- | --- | --- | --- | --- | --- | --- |
| Shannon | **0.021** | 0.390 |  |  |  |  |  |  |  |
| Fisher's alpha | **0.019** | 0.379 | **<0.0001** |  |  |  |  |  |  |
| Chao1 | 0.075 | 0.352 | **<0.0001** | **<0.000** |  |  |  |  |  |
| Bdellovibrionaceae | 0.218 | **0.008** | **0.012** | **0.019** | 0.166 |  |  |  |  |
| Mycobacteriaceae | 0.992 | 0.309 | 0.885 | 0.679 | 0.500 | 0.113 |  |  |  |
| Nocardiaceae | 0.422 | 0.503 | 0.283 | 0.183 | 0.219 | 0.468 | **<0.0001** |  |  |
| Caulobacteraceae | 0.996 | 0.863 | 0.610 | 0.571 | 0.262 | 0.797 | 0.475 | 0.993 |  |
| Nevskiaceae | 0.978 | **0.012** | 0.658 | 0.755 | 0.893 | 0.115 | 0.617 | 0.662 | 0.664 |
| Rhodospirillales Incertae Sedis | 0.296 | 0.359 | 0.350 | 0.300 | **0.031** | 0.938 | 0.704 | 0.985 | 0.034 |

**Figure S7**. Relationships based on the GLM results for the diversity indices in the biofilm communities as a function of the experimental factors (SS and %PS) and the relative abundance of the dominant taxa. See Table 2 for the complete GLM output; observe that not all significant predictors are included in the plots.


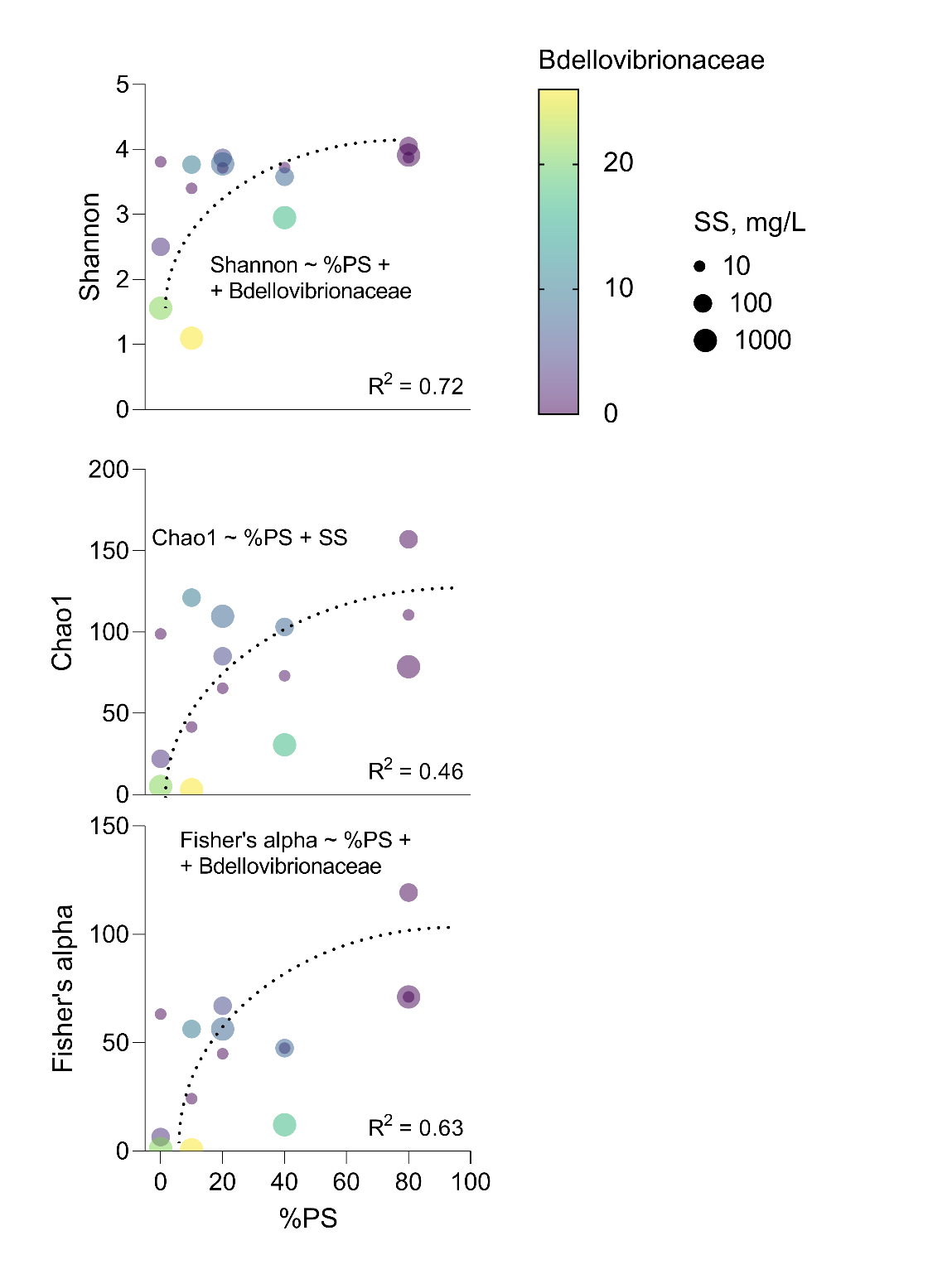


**References**

Blott, S.J., Pye, K., 2001. GRADISTAT: a grain size distribution and statistics package for the analysis of unconsolidated sediments. Earth Surface Processes and Landforms 26, 1237–1248. <https://doi.org/10.1002/esp.261>

Folk, R.L., Ward, W.C., 1957. Brazos River Bar: A Study in the Significance of Grain Size Parameters. Journal of Sedimentary Petrology 27, 3–26.

Gerdes, Z., M. Hermann, M. Ogonowski, and E. Gorokhova. 2019. A novel method for assessing microplastic effect in suspension through mixing test and reference materials. Scientific Reports 9. <https://doi:10.1038/s41598-019-47160-1>

Gordon, A.K., Palmer, C.G. 2015. Defining an exposure–response relationship for suspended kaolin clay particulates and aquatic organisms: Work toward defining a water quality guideline for suspended solids. Environmental Toxicology and Chemistry 34, 907–912. <https://doi.org/10.1002/etc.2872>

Konert, M. & Vandenberghe, J. 1997. Comparison of laser grain size analysis with pipette and sieve analysis: A solution for the underestimation of the clay fraction. Sedimentology, 44, 523–535, <https://doi.org/10.1046/j.1365-3091.1997.d01-38.x>.

Lundstedt, T., Seifert, E., Abramo, L., Thelin, B., 1998. Experimental design and optimization. Chemom. Intell. Lab. Syst. 42, 3–40.

Ramaswamy, V., Rao, P.S. 2006. Grain Size Analysis of Sediments from the Northern Andaman Sea: Comparison of Laser Diffraction and Sieve-Pipette Techniques. Journal of Coastal Research, 224, 1000–1009, <https://doi.org/10.2112/04-0162.1>.

Serra, T., Colomer, J., Cristina, X.P., Vila, X., Arellano, J.B., Casamitjana, X., 2001. Evaluation of Laser In Situ Scattering Instrument for Measuring Concentration of Phytoplankton, Purple Sulfur Bacteria, and Suspended Inorganic Sediments in Lakes. Journal of Environmental Engineering 127, 1023–1030. <https://doi.org/10.1061/(ASCE)0733-9372(2001)127:11(1023)>
